# Walking to a number: is there affective involvement in generating the SNARC effect in numerical cognition?

**DOI:** 10.3389/fpsyg.2024.1384818

**Published:** 2024-05-06

**Authors:** Hanna Segal, Joseph Tzelgov, Daniel Algom

**Affiliations:** ^1^School of Psychological Sciences, Tel-Aviv University, Tel Aviv, Israel; ^2^Shaanan Academic Teachers College, Haifa, Israel; ^3^Department of Psychology, Ben-Gurion University, Be'er Sheva, Israel; ^4^Achva Academic College, Arugot, Israel

**Keywords:** affective-SNARC, polarity-correspondence, spatial effects, body motion, emotion

## Abstract

The effect known as the spatial-numerical association of response codes (SNARC) documents fast reaction to small numbers with a response at the left and to large numbers with a response at the right. The common explanation appeals to a hypothetical mental number line of a left-to-right orientation with the numerical magnitudes on the line activated in an automatic fashion. To explore the possibility of emotional involvement in processing, we employed prototypical affective behaviors for responses in lieu of the usual spatial-numerical ones (i.e., of pressing lateralized keys). In the present series of experiments, the participants walked toward a number or walked away from a number (in a physical approach-avoidance setup) or said “good” or “bad” in response to a number. We recorded strong SNARC effects with affective responding. For example, it took participants longer to say “good” than “bad” to small numbers, but it took them longer to say “bad” than “good” to larger numbers. Although each particular outcome can still be accounted for by a spatial interpretation, the cumulative results are suggestive of the possibly of affective involvement in generating the effect.

## 1 Introduction

The Spatial-Numerical Association of Response Codes (the SNARC effect; Dehaene et al., 1993) is over a quarter of century old. Within that span, the effect has evolved into arguably the single most popular phenomenon in current numerical cognition research (see Wood et al., 2008; Fischer and Shaki, 2014, for reviews). When people decide the parity (odd, even) of a number, they respond faster to numerically small numbers with a key on the left than with a key on the right, but they respond faster to numerically larger numbers with a key on the right than with a key on the left. Despite the voluminous research, a fully consensual explanation has eluded students of the SNARC effect. In the most widely accepted account (e.g., Dehaene, 1997), the influence of response laterality is explained by the key notion of a left-to-right extending mental number line with the numbers on the line activated in an automatic fashion. The omnipresent influence of numerical magnitude – even when the task does not involve magnitude – is taken to support the automatic activation of numerical values on the line.

Our point of departure in this study was the alternative account of the SNARC effect suggested by Proctor and Cho (2006) who focus on the congruity structure inherent in the SNARC task. Both the number dimension and the response dimension are marked by a positive pole (large number, right side) and a negative pole (small number, left side). These poles can correspond (e.g., when a large number is responded by a right-hand key) or conflict (when a large number is responded by a left-hand key) on any given trial. Polarity correspondence is conductive to fast responses, whereas polarity mismatch results in slow responses; this difference generates the SNARC effect. In Proctor and Cho’s (2006) account, the notions “positive pole” and “negative pole” or “+polarity” and “-polarity” are neutral terms. Here we wished to test the idea that these notions are genuinely positive and negative, namely, that they carry emotional valence (however slight; see Lebrecht et al., 2012 on “micro-valences”).

For a working hypothesis, we conjectured that small numbers are “bad” and that larger numbers are “good”. In the dictionary, “more” is associated with positive terms such as greater and better, whereas “less” is associated with negative terms such as unimportant or diminished (see both The American heritage dictionary, 1985; Merriam-Webster, 2007). In English, Hebrew, and other languages, values of highly abstract concepts, including the linguistic structure of exact pure numbers (natural numbers), are often associated with positive and negative valence (Goldstone and Barsalou, 1998; Barsalou, 1999; Casasanto, 2009). The associations arise and are maintained through a wealth of metaphorical expressions in language and in culture at large (Lakoff and Johnson, 1980, 1999). We expected to witness the affective associations via approach and avoidance responses – likely the oldest distinction in the analysis of behavior (Elliot, 2006; Wilkowski and Meier, 2010). Barsalou, Casasanto, and their associates provide ample evidence for the perceptumotor expressions and mappings of the most abstract constructs (see also, De la Fuente et al., 2015). Consequently, the hypothesis is forwarded that the SNARC effect derives from an emotionally charged congruity structure between number-valence and side-of-response-valence.

To test our emotion hypothesis of the SNARC, we introduced *affective responses* into the SNARC task. Our participants were walking to a number or were moving away from a number (i.e., they were actually approaching or avoiding the stimulus screen) or were saying “good” to a number or “bad” to a number, as a function of the number’s magnitude. If numbers carry emotional valence, people should avoid small “bad” numbers more swiftly than large “good” numbers. Conversely, they should approach large “good” numbers faster than small “bad” numbers. These predictions were borne out by the results. To anticipate, our results revealed that affective responses to numerical magnitude (smaller or larger than 5) generated typical and rather strong SNARC effects.

However, to fully appreciate the meaning of our results, we first refer to four relevant investigations. The first by Gevers et al. (2010) showed that the SNARC effect can be produced by verbal responding. The second by Leth-Steensen and Citta (2016) first tested the hypothesis that the verbal responses “left” and “right,” used by Gevers et al. (2010), stand for “bad” and “good”, respectively. The third, the recent study by Mirabella et al. (2023), showed that whole-body movements, used in the present study, comprise an ecologically valid model for emotional responses as expressed via approach-avoidance reactions. The fourth study, by Mourad and Leth-Steensen (2017) argues against too firm a conclusion with respect to an exclusive affective explanation; the authors submit that an explanation in terms of spatial representation is still possible.

### 1.1 Affective responses and SNARC: four recent studies

The most notable feature of the study by Gevers et al. (2010) was the dumping manual responding in a SNARC task. The authors replaced manual responding with the *verbal* responses, “left” and “right.” Gevers et al. (2010) found an appreciable verbally-derived SNARC effect, which was comparable with the SNARC effect produced by the typical manual responses. Notice though that the verbal responses, left–right, still retain the spatial framework of the original SNARC.

Subsequently, Leth-Steensen and Citta (2016); see also Leth-Steensen et al. (2011) attempted yet another vocal SNARC study. Some of their participants used the words “bad” and “good,” whereas others used the previous “left” and “right.” The authors contemplated the present hypothesis, to wit, “it could be assumed that bad would naturally be coded as –polarity and good as +polarity, [so that] an analogous SNARC-like effect (i.e., faster bad responses for smaller numbers and faster good responses for larger numbers) [is] predicted” (Leth-Steensen and Citta, 2016, pp. 484–485). The authors did not find a SNARC effect using bad-good although they did find a small effect using left–right. We thought it prudent to invest in another attempt at verbal SNARC and arguably at affective SNARC – with the affective responses expressed by bad-good words and by whole-body movements.

Concerning whole-body movements, the recent study by Mirabella et al. (2023) provides support for employing motion by the whole body (approach-avoidance) as a means for assessing affective engagement. The stimuli were angry, happy, or neutral facial expressions with the participants stepping forward upon a go-signal when an emotional face was presented. Both reaction times and errors were larger with angry faces than with happy faces. Over and above this particular association between approach-avoidance and face-valence, again, the Mirabella et al. (2023) study provides powerful support for body locomotion as a means for assessing affective processing (see also Chajut et al., 2010).

The Mirabella et al. (2023) study reported a further notable result. When the task was gender classification of the face, face-valence did not affect the responses. The authors concluded that the link between emotional valence and approach-avoidance is not automatic; rather, the stimuli and responses are consciously appraised with performance depending on the goal and the prevailing context (see also, Marsh et al., 2005; Mirabella, 2018; Mancini et al., 2020). Now, the human face is an incredibly richly nuanced stimulus so that the identification of conscious appraisal by the authors is well taken. However, the evolution-honed human predisposition to approach positive stimuli and avoid negative stimuli, subsumed under *the fundamental hedonistic principle* (Higgins, 1997), concerns stimuli essential for survival. People approach food, water, and partners, and avoid pain, poison, or predators. Conscious appraisal with respect to these stimuli is not realistic simply for lack of any latitude.

The fourth and final study by Mourad and Leth-Steensen (2017) offers an alternative explanation to the present affective approach-avoidance scheme, for the SNARC effect. The authors argue that the vast majority of SNARC studies are performed within a horizontal-horizontal spatial frame of reference. Consider the standard setup: The participant responds to a property of the single number presented (e.g., smaller, or larger than 5) by pressing one of two lateral keys. When this arrangement is coupled with the theory of a horizontal mental line, the result is compatibility between a left–right subjective number placing and a left–right response placing. How vital is the horizontal-horizontal spatial reference frame for generating the SNARC effect? Mourad and Leth-Steensen (2017) showed that it is vital. Espousing the Mourad-Leth-Steensen scheme, stepping forward toward a number or stepping backward away from the number comprise proximo-distally aligned responses. If the participants imagine a similar near-far spatial frame of reference for the numerical magnitudes presented, then the alignment of spatial reference frames suffices to generate the SNARC effect. This explanation retains the original spatial flavor of the SNARC.

### 1.2 The present study: affective responding to numbers in the SNARC experiment

Our goal in this research was to test the emotion hypothesis of the SNARC effect. We surmised that it is plausible that numbers carry emotional valence or microvalence (Lebrecht et al., 2012) so that people tend to react to small numbers with avoidance of the kind associated with negative stimuli (e.g., snake, poison) and react to large numbers with the same approach behavior associated with positive stimuli (e.g., food, water). We further used compelling evidence that left and right responses carry negative and positive valence, respectively (Casasanto, 2009). Given the standard SNARC task of deciding the parity (odd, even) or magnitude (small, large) of the presented number, a congruity structure is created between the valence of the number and the valence of the response. The values of valence can match or mismatch, which creates the SNARC effect.

Five experiments tested our emotion theory. The task for the participants was speeded classification of numerical magnitude. Presented with a single digit, the participant decided, while timed, whether it was larger or smaller than 5. The unique feature of the present experiments was the nature of the response. Rather than pressing lateralized keys (as in the original SNARC study) or saying aloud the spatial terms “left–right” (Gevers et al., 2010), our participants responded by saying “good” or “bad” to the number or by *physically* approaching or avoiding the number. If numbers carry emotional valence, people should avoid “bad” numbers (i.e., small numbers) more swiftly than “good” numbers (i.e., large numbers), but they should approach “good” numbers faster than “bad” numbers. The same pattern should emerge for the verbal responses “good” and “bad.”

## 2 Experiment 1: walking to a number

In this experiment, the participants were physically walking toward a number or were walking away from a number. The task was speeded classification of numerical magnitude. The participants decided whether the numeral, presented on a monitor mounted in front of them, was larger or smaller than 5. Notably, the participant indicated the decision by walking toward the number or by walking away from the number. In one condition, the participants responded to large numbers by approaching them and to small numbers by retreating away from them. In another condition, the participants responded in the reverse regime: They approached the stimulus if it was a small number, but avoided the stimulus if it was a large number. If numbers carry emotion, then approach responses should be fast to large numbers and avoidance responses should be fast to small numbers more than vice versa. This hypothesis was tested in Experiment 1.

### 2.1 Methods

#### 2.1.1 Participants

Twenty young students (11 males) from the Ort-Tivon High School of Kiryat Tivon volunteered to perform in the experiment within the framework of a scientific project. They were between 16 and 18 years of age and all had had normal or corrected-to-normal vision. All of the participants were naïve with respect to the purpose of the experiment. This and the subsequent experiments were approved by the ethics committee of the School of Psychological Sciences at Tel-Aviv University. Sample size in this and the subsequent experiments was decided based on the accepted number of participants in relevant studies in the field (e.g., Zohar-Shai et al., 2017). In the recent study by Mirabella et al. (2023) with a similar structure and number of variables, the sample size was 20, sufficient to obtain a power of 0.80 for the analyses. We employed at least 20 participants in each of the experiments with 32 participants in Experiment 3.

#### 2.1.2 Apparatus and stimuli

We used a commercial dance mat for an electric platform (110 cm × 90 cm, Dance-Dance-Revolution Super Deluxe Pad product). The pad was hooked up with an LG Pentium computer (through its game port) with synchronization (and all other event timing) governed by a directRT Precision Timing Software (Version 2008.1.0.11). Time resolution of this system was 8 ms on average (on a par with the typical resolution for standard key pressing). The stimuli were displayed on the grayish background of a 10 in. flat-screen color monitor (with an 1.6 GHz refresh rate, set at a resolution of 1,024 pixels × 600 pixels) mounted on the wall facing the participant.

The participant stood at the central position of the pad, facing the screen, which was placed at the longer end of the rectangular pad. The screen was mounted at the participant’s eye level, approximately 1.2 m from the face. A single number appeared on the screen and remained present until the participant’s (dominant) leg touched the pad at the adjacent position in front of the starting position or behind it. This duration (from stimulus onset to completion of the stepping) served as the main dependent variable. The participant then returned to the starting position, and the next trial began after 2 s.

The stimuli were the eight digits, 1, 2, 3, 4, 6, 7, 8, 9, presented singly on the screen. The digits appeared in Ariel, font size 86, to make them easily visible from the distance of 1.2 m. The digits were presented at equal frequency. The order of stimulus presentation was random and different for each participant.

There were two blocks of 48 trials, with each digit presented 6 times. In one block, the participants stepped forward when they detected a large number (i.e., larger than 5), but stepped backward when they detected a small number (smaller than 5). In the other block, the participants stepped forward when they detected a small number and stepped backward when they detected a large number. The order of blocks was random and different across participants. The participants performed eight practice trials prior to the experiment.

#### 2.1.3 Procedure

**Figure 1:**
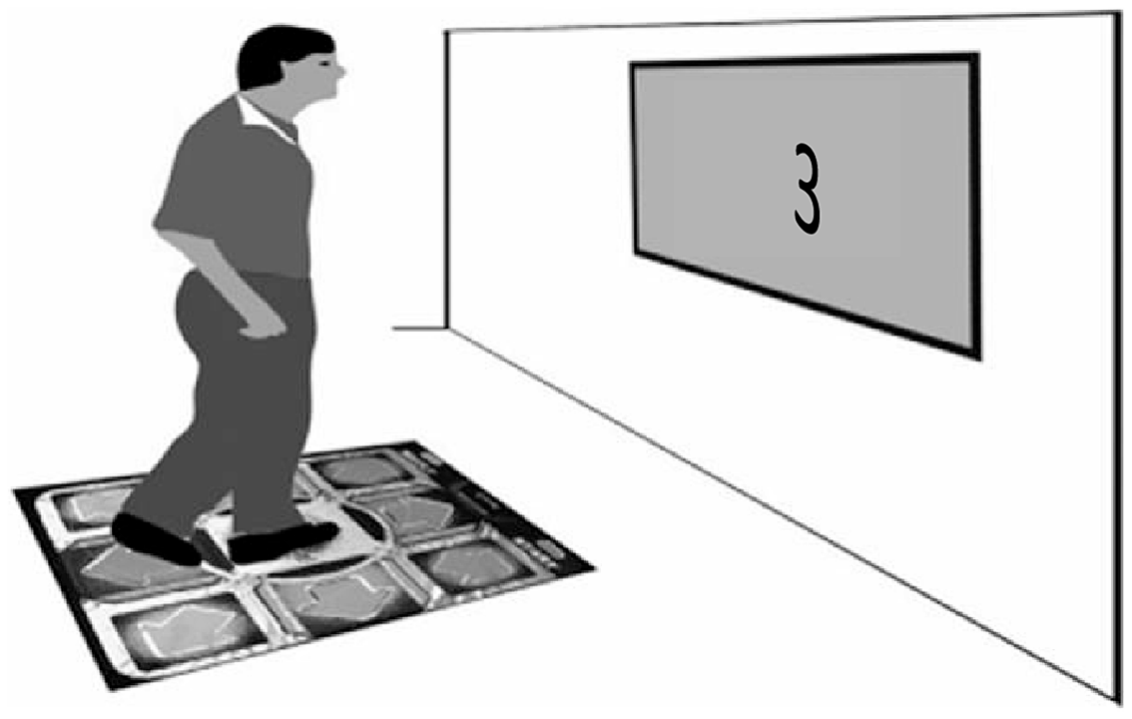
The setup of Experiment 1: The participant stepped forward or stepped backward in response to numerical magnitude.

The participants were tested individually in a dimly lit room. Each participant performed in two blocks, doing the walking 96 times in all. There was a break of 1.5 min between the blocks. In Figure 1, we illustrate the experimental task.

#### 2.1.4 Data analysis

Errors occurred predominantly due to the sensitivity of the pad to small movements that activated more than one region. Genuine errors were few at 1.5% and did not differ across conditions, *F* < 1. In addition to error, we excluded from the RT analyses responses longer than 2,200 or shorter than 250 ms (1.7%). There was not a time-accuracy tradeoff; the correlation between RT and error was 0.023 (*p* > 0.05).

### 2.2 Results and discussion

**Figure 2:**
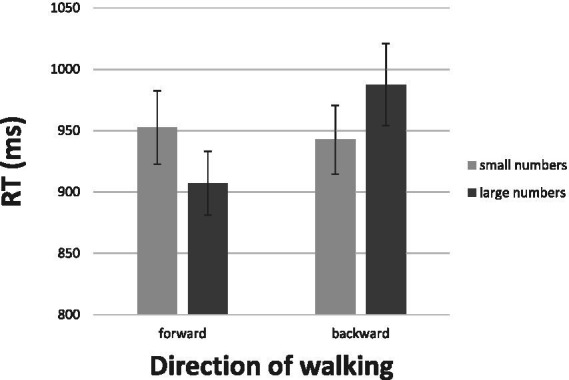
The results of Experiment 1: Mean RTs for walking forward and walking backward to a number as a function of its numerical magnitude. The bars depict one standard error around the means.

Figure 2 gives the results. Plotted are the mean RTs for the four experimental combinations: stepping forward to small numbers, stepping forward to large numbers, stepping backward to small numbers, and stepping backward to large numbers. Consider first the results for the *approach* (forward) responses at the left-hand half of Figure 2. Apparent is the difference in RT favoring large numbers: The latency of approaching large numbers was 907.04 ms on average, whereas that for approaching small numbers was 952.49 ms on average. This difference of 45.5 ms was highly reliable, *t*(19) = 2.97, *p* < 0.00, *d* = 0.67. The *avoidance* (backward) responses on the right-hand half of Figure 2 revealed a complete reversal of the pattern observed for approaching. The latency of avoiding small numbers was 942.64 ms on average, whereas that for avoiding large numbers was 987.34 ms on average. This difference of 44.7 ms was also reliable, *t*(19) = 2.33, *p* > 0.05, *d* = 0.52. The interaction of numerical magnitude (small, large) and type of response (approach, avoidance) further documented the full reversal in the pattern of responding, *F* (1, 19) = 10.22, *p* <. 05, η^2^ = 0.35.

Recall the fundamental hedonic principle: It asserts that approach and avoidance are people’s prototypical responses to emotion stimuli. Consequently, the different patterns observed in Experiment 1 lend support to the idea that pure numbers are emotional stimuli. The direction of the interaction between magnitude and approach-avoidance supports the association of small numbers with negative valence and of large numbers with positive valence.

### 2.3 The affective SNARC effect (aSNARC) – with the MARC effect offset

When deriving the SNARC effect, one should not ignore the confound generated by the related MARC effect (the linguistic Markedness Association of Response Codes effect; Clark, 1969; for numbers in particular, see Berch et al., 1999; Nuerk et al., 2004; Tzelgov et al., 2013). The MARC effect is the finding that even numbers are responded faster with a key at the right and odd numbers with a key at the left. Our emotion account (i.e., even numbers are “good”, odd numbers are ‘bad’) applies with equal force to the MARC effect, too (see also, Berch et al., 1999; Proctor and Cho, 2006; Leth-Steensen and Citta, 2016). Now, a well-recognized problem is that the MARC effect makes it difficult to detect the SNARC effect. Consider the following example (Zohar-Shai et al., 2017): The numbers 8 and 9 are responded faster with the right hand and the numbers 1 and 2 are responded faster with the left hand (than in the opposite mapping) – the SNARC effect. Simultaneously, the numbers 2 and 8 are responded faster with the right hand and the numbers 1 and 9 are responded faster with the left hand (than the opposite mapping) – the MARC effect. As Berch et al. (1999) explained, the presence of odd and even subsets of numbers counteracts the downward trend of left–right dRT as mandated by the SNARC effect. Thus, the MARC effect reduces the SNARC effect, and, at times, eliminates it altogether (Tzelgov et al., 2013).

**Figure 3:**
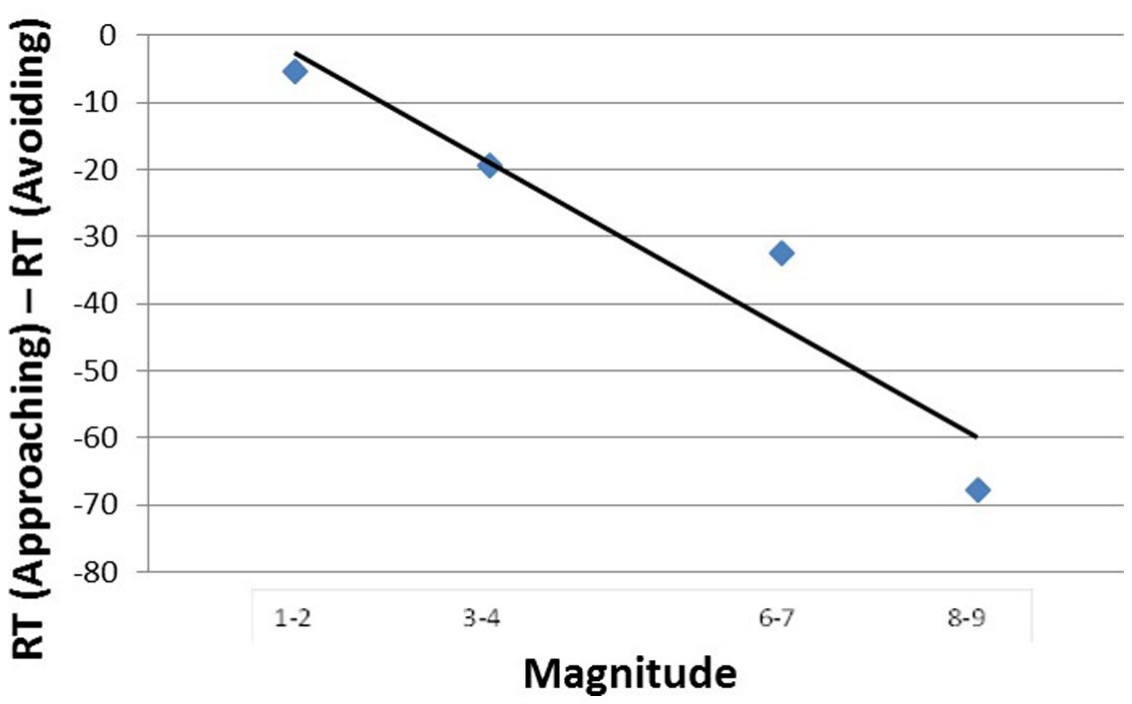
Mean dRT (the RT difference in ms. between approaching and avoiding the numbers in a bin) as a function of numerical magnitude.

One way to negate the influence of the MARC effect, starting already with Dehaene et al. (1993), is to plot dRT against bins of adjacent numbers (1–2, 3–4,…8–9) rather than against individual numbers (1, 2,…0.9). Because each bin entails an odd and an even number, the effect of MARC is eliminated by the opposing odd-even response asymmetries. At the same time, number magnitude is preserved by the bins. As a result, number magnitude becomes the predictor variable (as was initially suggested by Dehaene et al., 1993, and as is shown by the rigorous analysis by Tzelgov et al., 2013). This way of controlling for the MARC is particularly important in languages whose morphology enhances the MARC like Hebrew (the language of our participants). This tactic was successfully applied by Zohar-Shai et al., 2017 (for parity judgments, but it is also serviceable for judgments of magnitude in this experiment). In Figure 3 we present the affective SNARC effect derived in Experiment 1 (with the MARC controlled).

The negative slope of the function in Figure 3, −8.2, sustained by a high value of goodness of fit (*R*^2^ = 0.91, *p* < 0.05), can be taken to attest to the potency of emotion. Three points are notable. First, approaching-avoiding is not an arbitrarily chosen response mode – it is rather the prototypical, evolution-honed behavior when people face positive and negative stimuli. Second and pursuant to the previous point, the results may suggest that numbers carry emotional valence whose sign depends on their magnitude. Third, Experiment 1 lacks any affinity with a horizontal left–right orientation of numbers or with horizontal left–right responding (whether manual or oral).

Nevertheless, when appreciating the outcome of Experiment 1, one cannot rule out a spatial reference framework account as suggested by Mourad and Leth-Steensen (2017). Although the affective framework is suggestive, it is probably not the sole explanation of our results.

The results of Experiment 1 are novel and challenging, hence we deemed replication and extension invited.

In Experiments 2a, 2b, and 2c we tested affective verbal reaction to a number.

Following our hypothesis, saying aloud “left” and “right” (Gevers et al., 2010) is, in effect, a surrogate for values of valence: good and bad. If so, a SNARC effect should obtain merely by responding “good” or “bad” to a number. We note that Leth-Steensen and Citta (2016) did not find a SNARC effect with bad-good responses. However, given the results of Experiment 1, we decided to attempt another test of the idea. We expected people to respond “bad” more swiftly to small numbers than to large numbers, but to respond “good” more swiftly to large numbers than to small numbers.

## 3 Experiment 2a: saying “good” and “bad” to a number

### 3.1 Methods

#### 3.1.1 Participants

A new group of 20 young students from the Ort-Tivon High School of Kiryat Tivon and of Beit-Chaya High School of Kiryat Shmuel volunteered to participate in the experiment. They were between 15 and 18 years of age and all had had normal or corrected-to-normal vision. All of the participants were naïve with respect to the purpose of the experiment.

#### 3.1.2 Apparatus and stimuli

We used the same number stimuli as in Experiment 1. However, the participants no longer moved toward or away from the stimulus. They were sitting in front of a computer screen at a distance of approximately 60 cm. In order to avoid adaptation, we introduced a trial-to-trial spatial uncertainty of approximately 50 pixels around the center location. The participants responded *orally* by speaking the words “bad” or “good” into the microphone (Head Set Teac HPX-8 brand). Stimulus exposure was response-terminated. The interval between the participant’s response and the appearance of the next stimulus was 450 ms. The interstimulus interval was shorter than in Experiment 1 due to the different nature of the responses. As in Experiment 1, the digits were presented with equal frequency. The order of stimulus presentation was random and different for each participant.

There were two blocks of 48 trials, with each digit presented 6 times. In one block, the participant responded to a large number (i.e., larger than 5) by saying “good” and responded to a small number (smaller than 5) by saying “bad.” In the other block, the participant responded to a small number by saying “good” and responded to a large number by saying “bad.” The order of blocks was random and different across participants. The blocks were separated by a break of approximately 2.5 min.

#### 3.1.3 Procedure

The participants were tested individually in a dimly lit room. Presented with a number on the computer screen, the participant was asked to respond as quickly and accurately as possible by speaking the word “good” or “bad” into the microphone. A DirectRT software (Version 2008.1.0.11) recorded the time until the participant began to pronounce the response. Each participant performed in two blocks, with the eight numbers presented 6 times in a block, making for 96 experimental trials in all.

#### 3.1.4 Data analysis

The RT analyses included only correct responses. Technical errors were 3.1% and did not differ across conditions (*F* < 1). Errors in responding amounted to a minuscule 2.4% and they too did depend on condition (*F* < 1). Moreover, RTs shorter than 280 ms or longer than 2,250 ms (2.8% of all responses) were removed. There was not a time-accuracy tradeoff, with the correlation between RT and genuine error was not significant statistically (*r* = 0.134, *p* > 0.5).

### 3.2 Results and discussion

**Figure 4:**
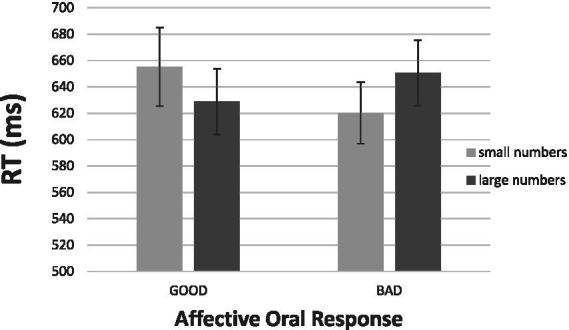
Results of Experiment 2a: Mean RTs for the verbal reactions “good” and “bad” to a number as a function of its numerical magnitude. The bars depict one standard error around the means.

Figure 4 gives the results. Salient to visual inspection is the presence of the expected interaction between the affective oral response (“good,” “bad”) and numerical magnitude (small, large). First consider the “good” responses at the left-hand half of Figure 4. It took participants 628 ms, on average, to respond “good” to a large number, but it took the same participants 655 ms, on average, to respond “good’ to small numbers. Although suggestive, this difference of 27 ms favoring large numbers was not significant statistically. For the “bad” responses at the right-hand half of Figure 4, we witnessed a reversal of the pattern observed with the “good” responses. The mean RT was 650 ms for saying “bad” to large numbers but the mean was 620 ms for saying “bad” to small numbers. This difference of 30 ms was highly reliable, *t* (19) = 2.51, *p* > 0.05, *d* = 0.56. Most important, the reversal of the pattern of responding was supported by a Numerical Magnitude × Response Valence interaction [*F* (1, 18) = 6.63, *p* < 0.05, η^2^ = 0.269].

**Figure 5:**
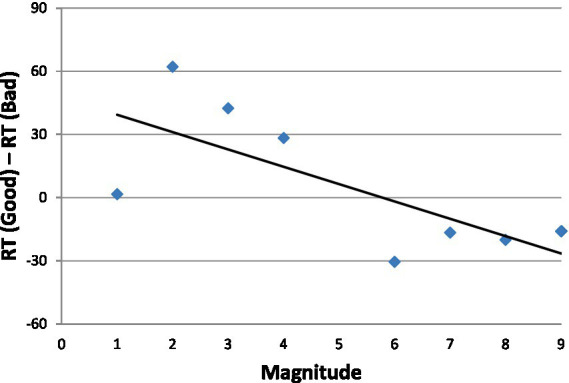
The aSNARC effect in Experiment 2a: Mean dRT (the RT difference in ms. between saying “good” and “bad” to the same numbers) as a function of the number’s magnitude.

In Figure 5 we present the aSNARC effect obtained in this experiment. We elected to present the data with the individual numbers in the abscissa (thereby ignoring MARC) to make aSNARC readily comparable to most SNARC renditions in the literature. Although less neat than Figure 3 (slope = −8.2, *R*^2^ = 0.51, *p* < 0.05) the function does show the dependence of subjective goodness on number magnitude.

Although the SNARC effect obtained with good-bad responses in Figure 5 is suggestive of affective involvement, we must note an alternative explanation that does not appeal to emotion. A glimpse at Figure 5 reveals grouping of the data into two distinctive categories: small numbers and large numbers. Therefore, it is possible that the classification, small vs. large [i.e., smaller or larger than 5] was driving the responses, with the labels good and bad simply associated with large and small, respectively.

One feature of the method in Experiment 2a was the close sequence of the two blocks with opposing instructions. The effect of emotion might be clearer in a design that bypasses such sequences. To test this possibility, while also providing a replication, we used the procedures of Experiment 2a with a single notable exception: The participant performed in the different blocks on separate days (see Zohar-Shai et al., 2017, for a similar tactic).

## 4 Experiment 2b: responding “good” and “bad” to a number on a different day

### 4.1 Participants

An independent group of 20 participants from the Beit-Rivka College of Education and from the Kiryat-Tivon High school performed in the experiment. They were between 15 and 22 years of age. Two did not return for DAY 2, so the data are based on the performance by 18 participants. All had normal or corrected-to-normal vision. All the participants were naïve with respect to the purpose of the experiment and performed in the experiment voluntarily.

### 4.2 Apparatus and stimuli

We used the same apparatus and stimuli as in Experiment 2a.

### 4.3 Procedure

The procedure was similar to that of Experiment 2a. However, the two different mappings of the responses (good, bad) onto numbers (small, large) were done on separate days. Our goal in employing this tactic was to reduce any carry-over or residual response conflict effects.

### 4.4 Data analysis

Errors amounted to 3.1% and did not differ across conditions (*F* < 1). For RTs, responses shorter than 280 ms or longer than 2,250 ms (3.2% of all responses) were removed. There was not a time-accuracy tradeoff; the correlation between RTs and errors was not significant (*r* = −0.22, *p* > 0.5). Our trimming procedures varied somewhat across experiments due to the differing distributions.

### 4.5 Results and discussion

**Figure 6:**
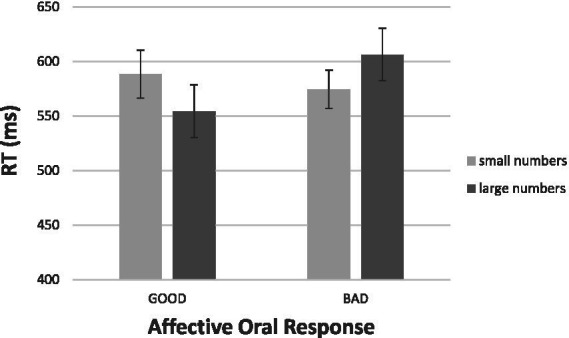
Results of Experiment 2b: Mean RTs for the verbal reaction “good” and “bad” to a number as a function of its numerical magnitude (mappings done on different days). The bars depict one standard error above the means.

First, consider the “good” responses at the left-hand half of Figure 6. Responding to a small number took longer than responding to a large number (588 and 554 ms, on average; *t* (17) = 1.93, *p* > 0.05, *d* = 0.45). This pattern reversed for the “bad” responses. Responding to a large number took longer than responding to a small number (606 and 575 ms, on average; *t* (17) = 1.65, *p* > 0.05, *d* = 0.39). The full reversal of the RT pattern was supported by the Numerical Magnitude x Response Valence interaction [*F* (1, 16) = 5.79, *p* < 0.05 η^2^ = 0.266].

**Figure 7:**
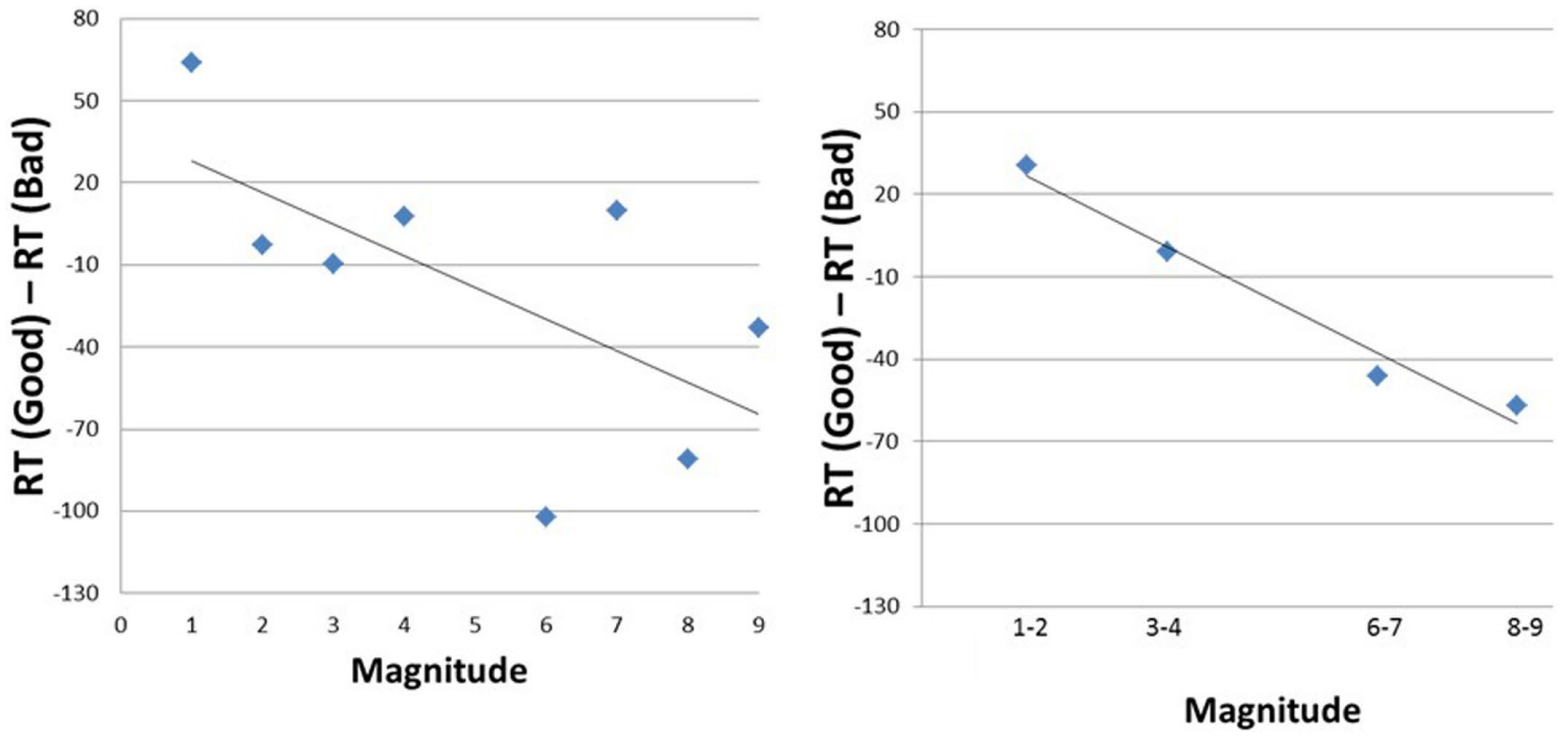
A pair of aSNARC functions without (left panel) and with (right panel) controlling for the MARC effect.

In Figure 7 we present two versions of the aSNARC function. The left-hand panel entails the usual description with individual numbers at the abscissa (i.e., with the MARC confound in force). The slope is steep at −11.5, but the goodness of fit is relatively poor (*R*^2^ = 0.41, *p* > 0.05). In the right-hand depiction, the MARC effect is removed through the two-digit bins at the abscissa, which resulted in a similar slope (−12.9) but with a greatly improved fit (*R*^2^ = 0.97, *p* < 0.05). Clearly the aSNARC is more visible when the effects of the MARC are removed.

The results of Experiment 2b reinforce the conclusions of Experiment 2a. People instinctively proffer a positive reaction to a large number but a negative reaction to a small number. The fact that the two different mappings of the responses were done on separate days reinforces the argument that numbers carry emotion and that the results are not a consequence of residual response conflict effects. Note that, with the current Figure 7, the alternative non-emotion explanation discussed in Experiment 2a is less compelling.

The results of Experiment 2b granted, we wished to attempt a replication with a single response regime per participant.

## 5 Experiment 2c: responding “good” or “bad” to a number with a single response per participant

In this experiment each participant performed in a single block of trials, entailing a single mapping of responses to numbers. One group responded “good” to large numbers and “bad” to small numbers, whereas another group responded “good” to small numbers and “bad” to large numbers. Supposedly, one group experiences conflict, whereas the other coherence. In this between-subjects design, competition at the response stage is ruled out because each participant performed in only one mapping.

### 5.1 Methods

#### 5.1.1 Participants

A group of 21 students from the Beit-Rivka College of Education performed in the experiment. They were between 20 and 23 years of age. All had normal or corrected-to-normal vision. All the participants were naïve with respect to the purpose of the experiment and performed in the experiment voluntarily. Each participant was assigned in a semi-random fashion into one of two groups defined by the response regime (10 participants performed under the mapping small-bad and large-good, and 11 under the mapping small-good and large-bad).

#### 5.1.2 Apparatus and stimuli

The same apparatus and stimuli used in Experiment 2a and 2b were applied again.

#### 5.1.3 Procedure

The procedure was similar to that of Experiment 2b, except for a single notable exception. In Experiment 2c, each participant performed using a single response mapping. In the first group (Mapping 1), the participant reacted by saying “bad” to small numbers and “good” to large numbers. In the second group (Mapping 2), the participant reacted by saying “good” to small numbers and “bad” to large numbers. In each condition/group, there were 48 experimental trials.

#### 5.1.4 Data analysis

Error amounted to 3.5% (including microphone/speech failures, 1.1%) and differed across conditions. The rate was 1.68% under the first regime and 5.1% under the second regime [*t* (19) = 2.29, *p* < 0.05]. For the RTs, responses shorter than 200 ms or longer than 2,000 ms (1% of the correct responses) were removed from the analysis. There was virtually no RT-accuracy tradeoff (Group 1: *r* = −0.021, *p* > 0.05; group 2: *r* = 0.06, *p* > 0.05).

### 5.2 Results and discussion

**Figure 8:**
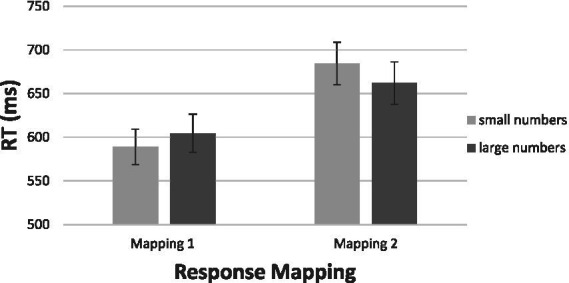
Results of Experiment 2c: Mean RTs for two mappings of “good” and “bad” responses to numbers as a function of numerical magnitude. [Group 1: Small = bad, and Large = good; Group 2: Small = Good, Large = bad].

The most revealing feature of the data in Figure 8 is the difference in average latency of responding between the two groups. The mean RT of the participants performing under the first regime was 597 ms, whereas the mean RT for those performing under the second regime was 673 ms [*t* (19) =2.41, *p* < 0.05]. The 76 ms difference favoring the first group likely resulted from the congruity entailed in Mapping 1 and the incongruity entailed in Mapping 2. In other words, Mapping 1 reflects the natural way that people respond to numbers, whereas Mapping 2 is counterintuitive. The interaction of stimulus magnitude (large, small) and response valence (good, bad) confirmed the reversal of preferential responding [*F* (1, 19) = 7.89, *p* < 0.01, η^2^ = 0.293]. Therefore, our data further documents the presence of a directional relation between numerical magnitude and affect. The interaction between numerical magnitude and saying “good” or “bad” is best understood by assuming that numbers carry valence as a function of their magnitude.

**Figure 9:**
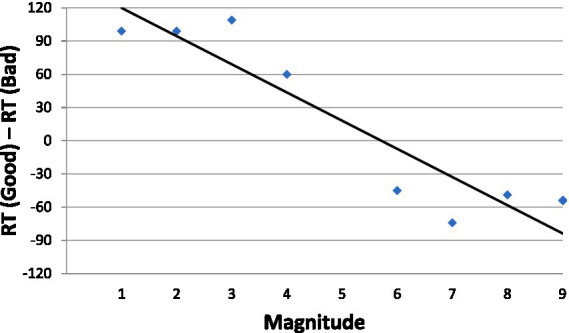
The aSNARC function derived across the two independent mappings of Experiment 2c.

The aSNARC function is presented in Figure 9. The function is impressive in view of the fact that it is based on two independent-group mappings of the responses to the same numbers (and without even removing the MARC). The slope of −25.5 supported with an *R*^2^ value of 0.86 is impressive, *p* < 0.001.

Close scrutiny of Figure 9 reveals a binary grouping of the data into small vs. large numbers, a feature observed already in the results of Experiment 2a. It is thus possible that the participants were actually classifying numbers into small vs. large with the designations good-bad merely associated with magnitude. We acknowledge this alternative non-affective account, although believe that it carries less weight with the current between-participant design. The huge difference between mappings is interpretative in terms of affection.

## 6 Experiment 3: walking to the color of a number

In the present experiment, the numbers appeared in different colors and the participants reacted to the *color* of the number rather than to its numerical magnitude. Therefore, number in this experiment was a *task-irrelevant* attribute. When presented with a number in color, the participant walked toward the number or walked backward away from the number depending on the *color* of the number. If emotion is activated in an automatic fashion, then the same interaction of approach-avoidance and numerical magnitude observed in the previous experiments should surface again.

Tzelgov (1997) proposed a distinction between intentional and autonomous processes in the SNARC effect. In our previous experiments, processing was intentional as numerical magnitude was part of the task requirement. In Experiment 3, by contrast, magnitude was not part of the task requirement, so that number magnitude was not processed in a conscious intentional mode. The task (of color classification) can be completed without recourse to the carrying number. Various biases have a smaller effect with the autonomous automatic processing used in Experiment 3 than with autonomous intentional processing used in Experiments 1–2.

Again, the task in Experiment 3 was responding to the color of the number. The participant indicated the color by walking toward the stimulus or by walking away from the stimulus. For example, some participants responded to the colors red and blue by approaching them and to the colors green and brown by retreating away from them, whereas other participants responded in the reverse regime. If numbers carry emotion, the approach responses should be faster to large numbers and avoidance responses should be faster to small numbers – despite the fact that magnitude is completely irrelevant to the task of color classification at hand.

### 6.1 Methods

#### 6.1.1 Participants

A group of 32 students from the Shaanan Academic Religious Teachers’ College of Haifa performed in the experiment. The participants volunteered to participate in response to an announcement in school. They were between 22 and 26 years of age and all had normal or corrected-to-normal vision participated. All of the participants were naïve with respect to the purpose of the experiment.

#### 6.1.2 Apparatus, stimuli, and design

We used the same commercial dance-mat for the electric platform. The stimuli were displayed on the grayish background of a flat-screen color monitor, mounted on the wall facing the participant, as in Experiment 1. The participant stood in the middle of the pad, in front of the screen placed at the longer end of the rectangular pad. The screen was placed at the participant’s eye level, approximately 1.2 m from the face. A single number appeared on the screen and remained present until the participant’s (dominant) leg touched the pad at the adjacent position in front of the starting position or behind it. This duration served as the main dependent variable. The participant then returned to the starting position, and the next trial began after 2 s.

The stimuli were the four digits, 2, 4, 6, 8, presented singly on the screen. We used only even numbers to rule out an influence of the MARC effect (e.g., Hines, 1990; Willmes and Iversen, 1995; Berch et al., 1999; Nuerk et al., 2004; Proctor and Cho, 2006; Zohar-Shai et al., 2017). Because the MARC depicts a difference between odd and even numbers regardless of magnitude, using only the latter eliminates the effect. At the same time, we retained the numerical magnitude necessary for the SNARC. Along with number bins (e.g., Experiment 1) this tactic is also often used to separate MARC and SNARC.

The digits were presented in one of the four colors: red, blue, brown, and green. Each of the four digits was presented three times in each of the four colors with equal frequency. The order of stimulus presentation was random and different for each participant. For each participant, two ink colors were assigned to an approach response (stepping forward upon detecting that color), and the remaining two were assigned an avoidance response (stepping backward upon detecting the color). The assignment of colors to the approach and avoidance responses was random and different across participants. There were 48 experimental trials, with each digit presented 12 times.

#### 6.1.3 Procedure

The participants were tested individually in a dimly lit room. Each participant did the walking task through 48 trials in all.

#### 6.1.4 Data analysis

Errors amounted to a minuscule 3.7% and did not differ across conditions (*F* < 1). For RT, responses shorter than 250 ms or longer than 2,500 ms (9.4% of all responses) were removed. As we mentioned, the small difference in cutoff points (across Experiments 1 and 3 using an electric platform) reflects small differences in the attendant RT distributions. There was not a time-accuracy tradeoff; the correlation between RT and error was not significant (*r* = −0.23, *p* > 0.05).

#### 6.1.5 Results and discussion

**Figure 10:**
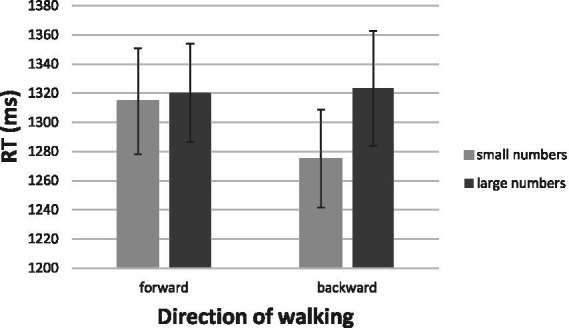
Speed of walking toward the color of a number or of walking away from the color of a number as a function of the number’s numerical magnitude. The bars depict one standard error around the means.

Figure 10 gives the mean RT for correct identification of the ink colors as a function of irrelevant numerical magnitude. Although the color-carrying numerals were irrelevant to color the task at hand, their magnitudes were noticed and impacted performance. In fact, the numerical values determined the speed of responses to color at each walking direction. Facing the color of a small number, the participants moved swiftly backwards, but moved more sluggishly forwards (means of 1,275 and 1,315 ms, respectively, [*t* (31) = 2.34, *p* > 0.05]). For large numbers, approach and avoidance responses were comparable (means of 1,323 and 1,320 ms, respectively, for moving backwards and forwards, [*t* (31) = 0.18, *p* > 0.05]). Despite the absence of a full reversal, the interaction of numerical magnitude (small, large) and type of response (backward, forward), *F* (1, 31) = 4.79, *p* < 0.05, η^2^ = 0.134, supported once again the possibility that numbers carry emotional valence.

The outcome of these analyses granted, we note that these results provide a weaker support for the emotion hypothesis than some of the other results. Numerically, the RTs for stepping backwards to small numbers were fastest, whereas the RTs for the other conditions were largely the same. We also note the weak statistical support for the aSNARC function below.

**Figure 11:**
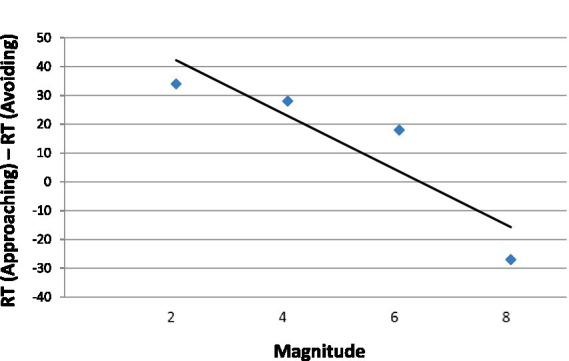
The aSNARC function for approach and avoidance of color as a function of task-irrelevant magnitude.

In Figure 11, we present the aSNARC effect for the present unusual situation in which numerical magnitude was irrelevant to the task at hand. Nevertheless, magnitude exerted a lawful influence on the affective responses to the colored numeral (slope = −9.7, *R*^2^ = 0.81, *p* > 0.05).

Numerical value influenced responding even when it was completely irrelevant to the task of color classification. Small numbers were responded to more swiftly by moving away from their color than by stepping forward, but this difference evaporated with large numbers. The overall pattern is consistent with the hypothesis that numbers carry emotional valence, the sign of which depends on their magnitude. This valence is activated in an automatic fashion as evinced by the lawful aSNARC function.

The results of Experiment 3 are not fully in harmony with the theory by Mirabella et al. (2023). According to that theory, the link between stimulus valence and motion is not automatic. When the task is goal-irrelevant, one does not expect to find a difference between approaching and avoiding. Here, we found an impact of stimulus valence even for irrelevant color. However, this impact was noticeably muted; it was much smaller than in the task-relevant motion in Experiment 1; also, we did not record an effect when approaching Experiment 3. Those features are consistent with Mirabella et al.’s idea. Further research should resolve this issue.

## 7 Conclusion

Our goal in this study was to raise awareness to the possibility of an affective involvement in numerical processing. This involvement is probably weak, at the level of micro-valence. Nevertheless, the association with affective values is systematic. Consequently, the unique feature of our experiments was the affective responses used. We pioneered the employment of emotion-laden affective responses in the study of the SNARC effect. Our participants walked toward a number (an approach response) or walked away from a number (an avoidance response), or simply pronounced “good” or “bad” to a number. We found that these affective responses were associated in a consistent fashion with the magnitude of numbers. Negative responses were particularly fast to small numbers and positive responses were particularly fast to large numbers.

According to the emotion hypothesis, retaining the spatial nature of the responses in SNARC studies, including verbal responses (left–right), is not necessary. Verbal responses that do not appeal to the concept of space can produce a SNARC effect if the requisite congruity structure – corresponding or conflicting valence-laden poles – is established. Thus saying aloud “good” and “bad” to large and small numbers, respectively, should be as effective as saying “right” and “left.” According to the emotion hypothesis, “left” and “right” may actually be surrogates for values of valence, good and bad (e.g., Casasanto, 2009). If supported, the hypothesis challenges the spatial-numerical account of the SNARC effect. According to the emotion hypothesis, the SNARC effect is not truly associated with numbers or even with space. It rather describes an emotion-congruity structure.

Challenging the traditional mental-line account of numerical processing, our hypothesis is consistent (although not same) with that of Reynvoet and his colleagues who also mount a challenge to the mental-line dominance (e.g., Sasanguie et al., 2014; Reynvoet and Sasanguie, 2016; Sasanguie et al., 2017; see also, Bar et al., 2019). Recently, Sella et al. (2020) found that numerical order judgments are determined by long-term learning rather than by involvement by a hypothetical mental number line.

Affective involvement in numerical processing is a new idea that received initial support in the present study. As with all new ideas, this study, too, leaves open several questions to be pursued in future studies. How does one dissociate the critical components responsible for the current effect? We plan several follow-up studies by way of controlling for non-emotion candidate variables. In one, we make use of the current stimuli and whole-body movements with a single notable exception: rather than approaching or retreating, the participants will make a left or a right movement in response to numerical magnitude. This setup preserves whole-body motion and the spatial milieu but not the emotionally loaded approach and avoidance motions. Distance from the emotion stimuli remains invariant. In another, we plan to use the present setup with non-emotion stimuli; for example, the participants respond with approach-avoidance to the shape, triangle or square, presented on the screen in front of them. Yet another idea comes from the recently published study by Montalti and Mirabella (2024): Let people approach-avoid “good” and “bad” facial expressions, once with the face covered by a mask and once without any cover. In this setup, one has accurate control for the presence of emotion (uncovered face) or the absence of emotion (masked face) with (almost) the same stimuli. Converging evidence to emerge from these and other studies is bound to isolate further the influence of affect in numerical processing.

## Authors’ Note

1. Small numbers can be beneficial when, for example, they stand for cookies and large numbers can be detrimental when they stand for misconduct. However, we were careful to focus on pure number (numbers onto themselves) – suns any representation (see Stevens, 1951). 2. Due to the between-participants design, the depiction in Figure 8 differs from the previous bar graphs but shows the same effect. The data and materials for all experiments are available at https://osf.io/bxkgm/?view_only=022c83e2a1034cf9bc2c0548eff26f49.

## Data availability statement

The datasets presented in this study can be found in online repositories. The names of the repository/repositories and accession number(s) can be found in the article/supplementary material.

## Ethics statement

The studies involving humans were approved by TAU Psychology ethics committee July 2017. The studies were conducted in accordance with the local legislation and institutional requirements. Written informed consent for participation in this study was provided by the participants’ legal guardians/next of kin.

## Author contributions

HS: Writing – original draft, Conceptualization, Data curation, Formal analysis, Investigation, Methodology, Project administration, Software, Validation, Visualization. JT: Writing – review & editing. DA: Conceptualization, Data curation, Formal analysis, Funding acquisition, Investigation, Methodology, Project administration, Software, Validation, Writing – original draft.
